# Multimodal breast cancer diagnosis using feature fusion and deep learning

**DOI:** 10.3389/fmed.2026.1877662

**Published:** 2026-07-21

**Authors:** Varun Malik, Tahani Alsubait, Mudassir Khan, Upasana Lakhina, Alaa Menshawi, Stuti Mehla, Loveleena Mukhija, Meteb Altaf, Leila Jamel

**Affiliations:** 1 Chitkara University Institute of Engineering and Technology, Chitkara University, Rajpura, India; 2 Department of Computer Science and Artificial Intelligence, College of Computing, Umm Al-Qura University, Makkah, Saudi Arabia; 3 Department of Computer Science, College of Computer Science, Applied College Tanumah, King Khalid University, Abha, Saudi Arabia; 4 Department of Computer Science and Engineering, National Institute of Technology, Delhi, India; 5 College of Computer and Information Sciences, Imam Mohammad Ibn Saud Islamic University (IMSIU), Riyadh, Saudi Arabia; 6 Panipat Institute of Engineering and Technology, Panipat, India; 7 Disability Research Institute, King Abdulaziz City for Science and Technology, Riyadh, Saudi Arabia; 8 Department of Information Systems, College of Computer and Information Sciences, Princess Nourah bint Abdulrahman University, Riyadh, Saudi Arabia

**Keywords:** cancer detection, clinical image analytics, cross-modal learning, image-based disease prediction, medical decision support

## Abstract

**Introduction:**

Breast cancer is one of the leading health problems in the world, and the challenge lies in the fact that its diagnosis at the earliest possible and accurate rate is the main factor to guarantee a successful patient outcome. The traditional deep learning (DL) frameworks usually utilize data from a single modality at a time and, therefore, are not capable of addressing the complexity and heterogeneity of the disease, particularly when data are unavailable or incomplete.

**Methods:**

To address these constraints, a multimodal breast cancer diagnosis model is presented that consists of attention-based transformers to achieve efficient modality specific feature extraction, the modified mantissa search (MMS) algorithm to remove irrelevant features, and the American zebra optimization (AZO) algorithm to dynamically and efficiently combine features. Final classification is then performed using a lightweight convolutional neural network (LCNN) to avoid compromising diagnostic accuracy.

**Results and Discussion:**

The proposed model is highly generalizable and resilient to missing modalities, achieving 98.958, 97.37, and 99.438% accuracy on Mammographic Image Analysis Society (MIAS), BreakHis, and combined multimodal datasets, respectively. These findings reveal their usefulness and strength in clinical diagnostic cases with a variety of imaging data.

## 1 Introduction

Breast cancer is the most prevalent cancer that is regularly diagnosed in women all over the world and the most common cause of cancer-related deaths (1). Although timely diagnosis greatly enhances prognosis, the disease's heterogeneous nature and its varied manifestations across individuals pose challenges. The traditional imaging systems like mammography, ultrasound, and magnetic resonance imaging (MRI) have proven to provide useful diagnostic information: mammography is extensively used in initial screening, ultrasound can be used in real time and is radiation-free and MRI has high contrast resolution (2–4). Although these are the strengths, there exist limitations in each modality. As an example, mammography is less effective in dense breast tissue, whereas ultrasound is operator dependent and MRI is costly and not widely available (5, 6). Artificial intelligence (AI), especially deep learning (DL), has proven to be an effective means of analyzing medical images to improve detection, reduce human error, and support clinical decision-making (7–9). Image enhancement, segmentation, and feature extraction are used to enable DL models to recognize patterns associated with malignancy across imaging modalities (10, 11). Convolutional neural networks (CNNs) have also demonstrated encouraging results in categorizing breast lesions based on hierarchical features learned from raw image data (12, 13). The combination of machine learning (ML) and DL with medical imaging has enabled profound diagnostic sensitivity and specificity (14, 15). Nonetheless, the majority of the current solutions are single-modal and, therefore, do not reflect the complexity of breast cancer. Recent multimodal models that seek to combine information across various imaging sources continue to have problems: they fail to perform well at global and modality-specific feature extraction, semantic inconsistencies, unable to deal with modalities missing effectively, and do not have dynamic weighting mechanisms that can be used to explain the varying diagnostic value of each modality (16–18). The findings indicate excellent treatment results on benchmark datasets, which indicate the practical potential and applicability of the model in the development of breast cancer screening and personalized healthcare methods (19, 20). However, no studies have been carried out so far that investigate the opportunities of different modalities to enrich breast cancer screening (21–26). This research introduces a powerful diagnostic model that combines histology and mammography images, extracts modality-specific features, and achieves efficiency and accuracy through optimized feature selection and lightweight classification. The attention models, which are transformer based, are applied to identify rich, hierarchical features that represent local and global information in histology and mammography images. The modified mantissa search (MMS) algorithm is a dimensionality-reduction feature search algorithm that eliminates redundant and irrelevant data to minimize computational complexity. To achieve successful multimodal feature fusion, particularly when features are missing, the American zebra optimization (AZO) algorithm is used to provide robust and dependable feature integration. Lightweight convolutional neural network (LCNN) is a fast and accurate classification model, and is ideal for real-time applications in resource-limited clinical environments. It is a powerful solution to issues in diagnosing breast cancer through a combination of smart feature extraction, smart selection, and efficient classification. Depending on the contributions, develop the research questions (RQs).

RQ1: Does the accuracy of detecting breast cancer increase when histology and mammography are combined?
RQ2: What effects do low- and high-level traits unique to a modality have on diagnostic performance?
RQ3: What effects does feature selection have on prediction accuracy and efficiency?
RQ4: Is it possible for a lightweight model to remain accurate when multimodal data is incomplete?

To address this gap, a multimodal breast cancer diagnosis model is proposed that uses both histopathology and mammography images to improve classification performance through optimal feature fusion and lightweight deep learning. The key contributions of the proposed model are given below:

1. To effectively capture detailed and comprehensive information from each modality, attention-based transformer models are employed. The models are adept at learning both low-level features (such as edges and textures) and high-level semantic features (such as cell or lesion patterns), which are specific to histology and mammography images.
2. The proposed model addresses the curse of dimensionality and redundancy in high-dimensional medical imaging data using the MMS algorithm. For effective multimodal fusion, especially when dealing with incomplete or missing modality data, the AZO algorithm is used.
3. The diagnosis and classification of breast cancer is performed using LCNN, which maintains high accuracy while minimizing computational cost, making it suitable for a real-time clinical environment. LCNN benefits from optimized, fused features, leading to improved predictive performance without the overhead of complex architectures.
4. The effectiveness of the proposed model is validated using two widely used benchmark datasets. The MIAS dataset contains mammographic images with various breast tissue densities and abnormalities, while the BreakHis dataset includes Histopathological breast cancer biopsy images at multiple magnification levels.

The rest of this article is organized as follows. Section 2 presents the review of literature on breast cancer diagnosis using artificial intelligence. Section 3 illustrates the proposed methodology, including feature extraction, feature reduction, optimal feature fusion, and classification. The results and comparative analysis of proposed and existing models are discussed in Section 4. The article concludes in Section 5.

## 2 Related studies

Youssef et al. (27) have proposed a hybrid feature extraction method that uses a DL model to improve breast cancer prediction. Support vector machine (SVM) and extreme gradient boosting (XGB) techniques achieve up to 96.22% accuracy, 97.19% sensitivity, and 95.23% specificity, showing potential for early breast cancer detection through thermal imaging. Maurya et al. (28) have proposed a modified Vision Transformer (Breast Cancer Classification using Lightweight Customized Vision Transformer [BMEA-ViT]), which replaces the original, notoriously computationally intensive multi-headed self-attention (MSA) with a multi-headed external attention (MEA) unit that achieves linear complexity and improves generalizability. Ruan et al. (29) have developed a diagnostic BI-RADS classification using B-mode images, Nakagami parametric images, and semantic features. Huang et al. (30) combined deep features with edge-focused information to improve segmentation accuracy using Edge-Aware Multi-Scale Group-Mix Attention Network (EMGANet), which efficiently integrates global and local features and enhances cancer border detection. Gezimati and Singh (31) have proposed DL-based multimodal breast cancer diagnosis and characterization system using terahertz (THz) and infrared (IR) imaging data. Rahman et al. (32) have proposed a bimodal sensing system that uses tactile and multispectral sensors to distinguish between tumors and non-tumor breast conditions. Cho et al. (33) have proposed a DL-based ultrasound image segmentation and classification approach for accurate breast cancer diagnosis. Kansal and Kansal (34) highlighted the critical need for disease prediction. Deng et al. (35) have presented the first distributed learning method [Federated Block Coordinate Descent/Federated Stochastic Block Coordinate Descent (FedBCD)] for collaborative breast ultrasound images, addressing data privacy and modality integration. Chikkala et al. (36) have introduced a bidirectional recurrent neural network (BRNN) for histopathology image analysis.

### 2.1 Problem description

Breast cancer is still a major worldwide source of anxiety, and in order to lower mortality and enhance patient outcomes, primary and correct recognition is critical. The problems of existing breast cancer diagnosis models are summarized in Table 1. Conventional DL-based diagnostic systems have largely relied on unimodal imaging data, such as histopathology or mammography (37–40). The clinical application is further hampered by the challenges of high data dimensionality (41), inefficient integration of complementary information, fixed modality weighting, and limited robustness to missing modalities (42–45). The model uses attention-based transformer networks to represent modality-specific high- and low-level features, making it modality-aware. The objectives below used to fill the research gaps.

- Multimodal diagnostic model combines both the mammography images and the histology, which appeals to the depth and the accuracy of the breast cancer detection.
- Each modality is captured at low- and high-level features, which ensures that its model makes the most out of the strengths of various data.
- The data is optimized in terms of dimensionality, and only the most relevant features are considered, which enhances the processing speed and the overall results of prediction.
- There is also a lightweight classification framework that can work with incomplete data, but the diagnostic outcomes are high.

**Table 1:** Review summary of ML/DL models on breast cancer diagnosis.

References	Methodology	Technique used	Dataset	Findings	Research gaps
Youssef et al. (27)	Early breast cancer prediction	Gabor filters, Canny edges, ResNet-50 and MobileNet	Thermal imaging	Accuracy 96.22%, sensitivity 97.19%	Limited to morphologic information about the lesion
Maurya et al. (28)	Breast cancer classification	External attention (MEA) for disease detection	BreakHis	Accuracy 95.74%−97.25%	Malignant lesions exhibit stiffer characteristics
Ruan et al. (29)	Ultrasound breast tumors diagnosis	Two-branch Transformer-CNN, feature fusion module	Multicenter BI-RADS 4 database	AUC 0.856 and accuracy 97.56%	The network had a limitation in not utilizing spatial information
Huang et al. (30)	Breast cancer image segmentation	EMGANet for edge feature and multi-scale attention	Dataset-B, BUSI, BUSI-WHU	Accuracy 98.56%, Mean IoU 90.32%	This model does not learn the unique features of each mode well.
Gezimati et al. (31)	Multimodal breast cancer characterization	THz and IR imaging, decision-level fusion	IR thermography dataset	Accuracy 96.6%, sensitivity 96.3%	An expensive multi-head self-attention model limits performance
Rahman et al. (32)	Bimodal profile for breast cancer detection	CNNs for feature extraction	Tactile and spectral datasets	Accuracy 83% for malignant tumors	The insufficient feature extraction ability creates high false positives
Cho et al. (33)	Breast cancer segmentation and classification	U-Net on B-mode and SE-mode Ultrasound	Clinical ultrasound data	Accuracy 98.46%, Dice score 78.23%	Data accessibility and the challenge of data imbalance.
Kansal et al. (34)	Multi-class breast cancer classification	DenseNet for feature fusion	BUSI ultrasound dataset	Accuracy 98.28%	Complexity and frequently unclear interpretations of decisions
Deng et al. (35)	Joint learning for breast cancer diagnosis	JUVIL model with temporal-spatial adapters	Distributed image and video data	Detection accuracy 95.56%	Limited by the data dimensionality issue, which causes high false alarm
Chikkala et al. (36)	Breast cancer diagnosis	BRNN with ResNet50, GRU, residual branch	BreaKHis	Accuracy 97.25% and precision 87%	Fine details were missed during the segmentation process

## 3 Materials and methods

**Figure 1:**
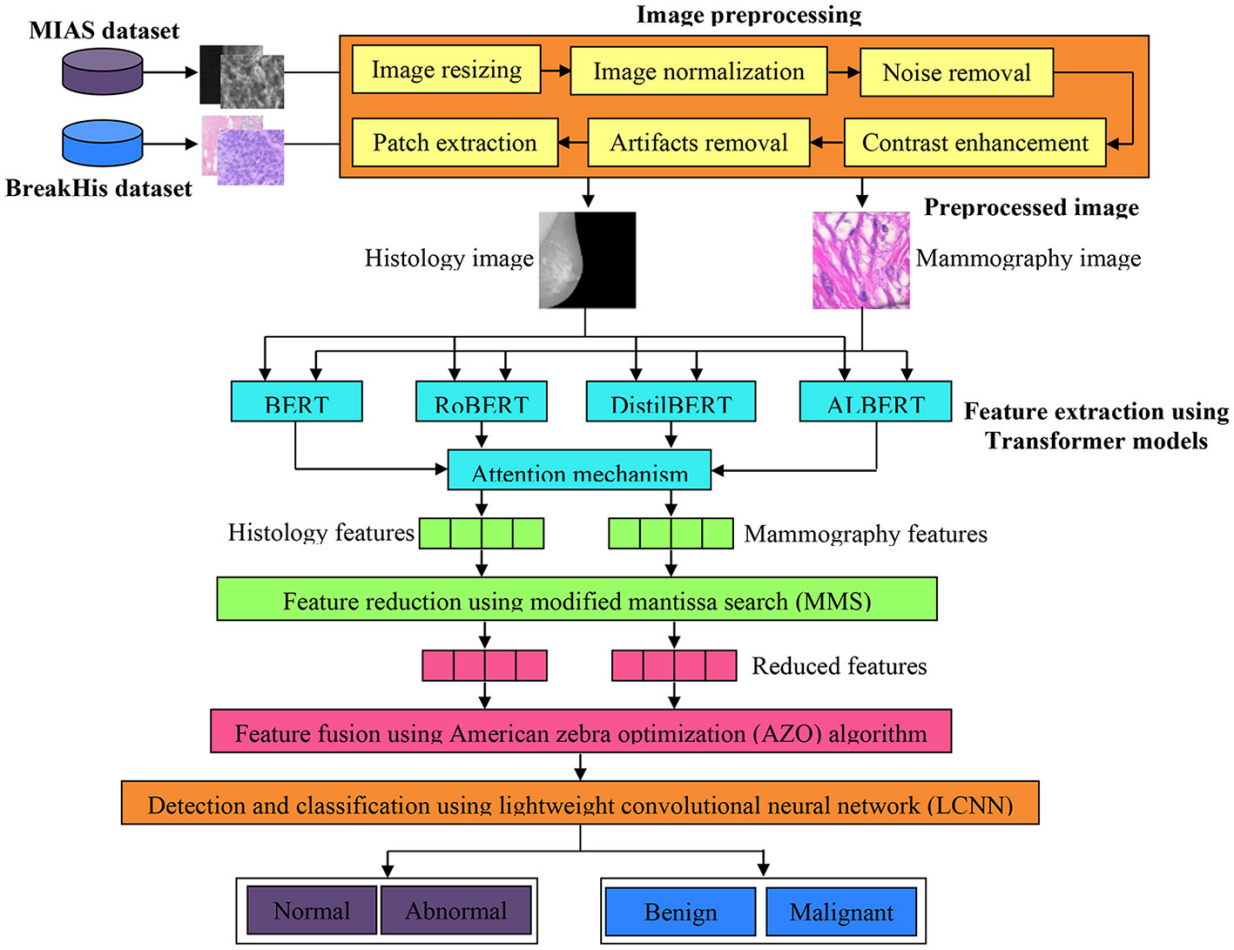
Optimal feature fusion and lightweight deep learning for multimodal breast cancer diagnosis.

Figure 1 presents the idea of optimal feature fusion and lightweight DL for multimodal breast cancer diagnosis using the MIAS and BreakHis datasets. The suggested model begins with the intensive image preprocessing phase to improve data quality and consistency across modalities. This involves image resizing (MIAS images resized to 299 pixels by 299 pixels and BreakHis histology images resized to 224 pixels by 224 pixels), image normalization to bring out uniformity in the intensity of the pixels, noise elimination, image contrasts to improve the visibility of tumor areas, artifact elimination to remove irrelevant distortions, and patch extraction to bring out important areas of interest.

### 3.1 Data preparation

**Figure 2:**
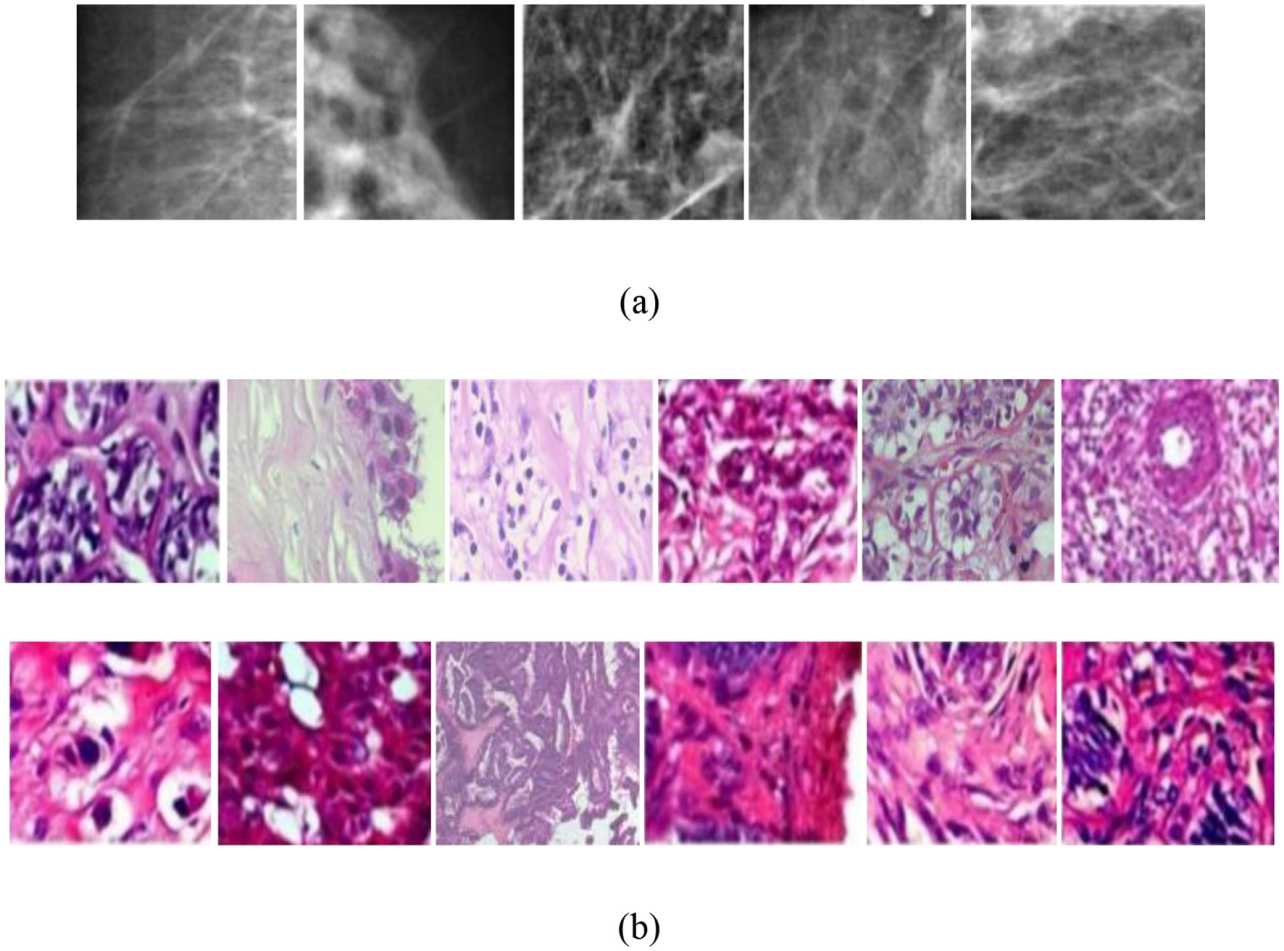
Representative sample images from **(a)** the MIAS dataset and **(b)** the BreakHis dataset; the MIAS dataset includes five classes. The BreakHis dataset includes 12 classes comprising both benign and malignant breast tissue types.

The proposed research use of an extensive multimodal dataset comprising histology and mammography images to improve the accuracy of breast cancer diagnosis. Histological data (46) were obtained by two popular open repositories, BreakHis and BACH, and mammography images (47). The data augmentation was used to overcome the imbalance between classes and enrich the training samples (Figure 2). The histology dataset was increased to 95,581 images (51,511 original and 44,070 augmented), and the mammography dataset was increased to 107,346 images (75,658 original and 31,688 augmented). Table 2 reports on the datasets and their sample information. Preprocessing of the image was conducted to align the dimensions of the input: 224 × 224 pixels in histology and 299 × 299 pixels in mammography to fit the DL models.

**Table 2:** Summary of class-wise distribution of original and augmented samples for histology and mammography datasets, along with training and testing splits.

S. No.	Class type	Number of samples			
		Original	Augmented	Training	Testing
BreakHis dataset					
1	Adenosis (A)	456	544	700	300
2	Benign (B)	100	400	350	150
3	Malignant carcinoma (DC)	2,749	2,251	3,500	1,500
4	Fibroadenoma (F)	1,127	1,373	1,750	750
5	*In situ* carcinoma (IS)	100	900	700	300
6	Invasive carcinoma (IV)	100	1,400	1,050	450
7	Lobular carcinoma (LC)	426	574	700	300
8	Mucinous carcinoma (MC)	495	505	700	300
9	Normal (N)	96	904	700	300
10	Papillary carcinoma (PC)	348	652	700	300
11	Phyllodes tumor (PT)	469	531	700	300
12	Tubular adenoma (TA)	630	870	1,050	450
	Total	6,096	10,904	12,600	5,400
MIAS dataset					
1	Normal (N)	800	700	1,050	450
2	Benign with calcification (BC)	750	750	1,050	450
3	Benign with mass (BM)	780	720	1,050	450
4	Calcification (CALC)	880	620	1,050	450
5	Mass (M)	894	606	1,050	450
	Total	4,104	3,396	5,250	2,250

### 3.2 Image preprocessing in histology and mammography images

The image preprocessing pipeline for histology and mammography images is meant to maximize the quality and consistency of the input data, which highly contributes to the model's performance.

- Resizing: to fit the input of deep learning models, both the histology and mammography images were resized to 224 × 224 and 299 × 299 pixels, respectively. The image size is also standardized, and the model processes all images in an orderly fashion (48).
- Pixel normalization: the intensities were normalized to the range of 0–1, which is used to normalize the image data and make the model training process more stable and quicker since the values are on an equal scale (48).
- Removal of noise, artifact: Gaussian filtering is used to remove noise and undesirable background data. It is essential to enhance classification accuracy, and avoid situations in which the model memorizes irrelevant features (49).
- Contrast enhancement: it is necessary to enhance the capacity of the model to evaluate minute information, such as tumors or lesions (50).
- Patch extraction: the patch extraction isolates a region of diagnostic interest (i.e., tumor regions, or regions with abnormal cell structure). It ensures that the model focuses on small but meaningful parts of the image, reduces the computational cost of processing the full image, and increases detection accuracy in critical regions (51).
- Data augmentation (flips, rotations): augmentation, for example, horizontal and vertical flips, and rotations can be used to combat class imbalance by artificially swelling the size of the dataset. In medical imaging, augmentation is essential, and the acquisition of large datasets across a variety of modalities may be challenging (52).

### 3.3 Feature extraction using attention-based transformer models

The transformer model that was adapted to the medical imaging analysis of multimodal breast cancer diagnosis was an attention-based transformer model, namely BERT (53), RoBERTa (54), DistilBERT (55), and ALBERT (56) in this research. Although these models are initially designed to be used with natural language processing (NLP) tasks, to learned in vision-based tasks with an intermediate representation process instead of direct raw image input or the application of standard Vision Transformers such as ViT or Swin Transformer. The image data (histopathology and mammography) were converted into structured and token-like sequences as part of the adaptation. First, the grid-based method was used to divide every image into fixed-size patches. Descriptive features were extracted for each patch using the following techniques: intensity histogram encoding, Gray-Level Co-occurrence Matrix (GLCM)-based texture descriptors, and local binary patterns. These feature vectors were subsequently encoded as one-dimensional (1D) sequences, which largely mimicked the token-embedding format used in NLP tasks. The bidirectional attention of BERT enabled it to better learn inter-patch dependencies, whereas RoBERTa achieved better generalization through training optimizations, such as dynamic masking. The simplified models DistilBERT and ALBERT were created to reduce the representational power and computational cost of the original model while retaining much of its representational power, making them especially applicable to scalable medical image analysis. Such cross-domain adaptation leverages the transfer learning capabilities of trained language models and is consistent with cases where annotated medical imaging data are scarce. These NLP-derived transformers can be trained on sizeable image datasets, unlike vision-specific transformers, which require large image datasets. Moreover, combined features of various imaging modalities were obtained by using a hybrid attention fusion mechanism (57–59). This combination improved the maintenance of modality-specific data and the model's sensitivity to minor pathological features, such as tumor margins and cell morphology, which are essential for early and precise diagnosis.

### 3.4 Feature reduction using the MMS algorithm

Deep learning-based medical image analysis is a process reduction, particularly when operating on high-dimensional data (extracted from multimodal data). This not only makes the computation process and the memory consumption much simpler, but also contributes to the enhancement of model generalization, decreases overfitting, and accelerates the training process. In that regard, feature reduction is achieved using the modified mantissa search (MMS) algorithm to optimize the massive feature vectors produced by transformer-based attention networks. MMS: it is a bio-inspired metaheuristic optimization algorithm that simulates the predatory behavior of mantissa, especially their adaptive selection of optimal routes to prey (60). The adapted version is more useful in its exploitation and exploration abilities by adding adaptive movement plans and opposition-based learning to balance local and global search. The MMS algorithm is used to determine the significance of each feature via a fitness function, typically associated with classification accuracy or information gain. It recursively chooses a set of features that maximize the separation between classes, unique between caring and malignant lesions in histology or abnormal vs. normal tissue in mammography. The algorithm produces a matrix of B solutions with dimensions (*P, C*), where *P* is the search space and *C* is the number of dimensions per solution. B solutions are generated to ensure that only the most relevant information is relayed to the classifier. In the s function, there is also a vector indicating the position of Mantissa *h*. On the edge of the optimization problem denotes in Equation 1, a random initialization is employed on a vecto (61).


$$\begin{array}{l} {\vec{p}}_{h}^{s}={\vec{p}}^{Ln}*\left(1-\vec{RV}\right)+\vec{RV}*{\vec{p}}^{Un} \end{array}$$ (1)

Where *Rv*_1_, *Rv*_2_, *Rv*_6_, *Rv*_7_, *Rv*_9_, *Rv*_10_, *Rv*_12_, *Rv*_13_, and *Rv*_17_ random values gotten by optimal solutions. ${\vec{p}}^{Ln}$ is the lower and ${\vec{p}}^{Un}$ is the upper bound of the *j*-dimension. The predator in search of prey can be evaluated scientifically as follows (62) in Equation 2:


$$\begin{array}{r} {\vec{p}}_{h}^{s+1}=\\ \{\begin{array}{c} {\vec{p}}_{h}^{s}+\left({\vec{p}}_{h}^{s}-{\vec{p}}_{m}^{s}\right)*{\vec{\tau }}_{1}+|\vec{\tau }|*\vec{u}*\left({\vec{p}}_{m}^{s}-{\vec{p}}_{n}^{s}\right),\\ if\;Rv_{1}\leq Rv_{2}\\ \vec{u}*{\vec{p}}_{h}^{s}+\left({\vec{p}}_{m}^{s}+\vec{Rv_{3}}*\left({\vec{p}}_{n}^{s}-{\vec{p}}_{d}^{s}\right)\right)*\left(1-\vec{u}\right),\\ otherwise \end{array} \end{array}$$ (2)

where $\vec{Rv_{3}}$, $\vec{Rv_{4}}$, $\vec{Rv_{5}}$, $\vec{Rv_{8}}$, and random vectors drawn from a uniform distribution between zero and one. Also, the number vector generated by the Lévy-Flight represents |τ_2_| arbitrary numeral result of normal dissemination with a kind of zero and regular deviation of one in Equation 3.


$$\begin{array}{l} \vec{u}=\{\begin{array}{l} 0\;if\vec{Rv_{4}}<\vec{Rv_{5}}\\ 1\;otherwise \end{array} \end{array}$$ (3)

Randomly ${\vec{p}}_{mr}^{s}$ selected solutions from the archive are sorted to determine the *h*th mantissa location (63) in Equation 4.


$$\begin{array}{r} {\vec{p}}_{h}^{s+1}={\vec{p}}_{mr}^{s}+\mu *\left(2*Rv_{7}-1\right)\\ {}*\left({\vec{p}}^{L}+\vec{Rv_{8}}\times \left({\vec{p}}^{U}-{\vec{p}}^{L}\right)\right) \end{array}$$ (4)

The bunkering behavior of mantissas and their prey is described as follows (64) in Equation 5:


$$\begin{array}{l} {\vec{p}}_{h}^{s+1}=\{\begin{array}{l} {\vec{p}}_{h}^{s}+\alpha *\left({\vec{p}}_{mr}^{s}-{\vec{p}}_{m}^{s}\right),ifRv_{9}\leq Rv_{10}\\ {\vec{p}}_{mr}^{s}+\mu *\left(2*Rv_{7}-1\right)*\left({\vec{p}}^{L}-\vec{Rv_{8}}\right)\\ \times \left({\vec{p}}^{U}-{\vec{p}}^{L}\right)),otherwise \end{array} \end{array}$$ (5)

Where $p_{h,g}^{s}$ are *h*th represents the current position of the *g*th dimension of the mantissa in Equation 6.


$$\begin{array}{l} f=1-s\%\left(S_{a}/X\right)/\left(S_{a}/X\right) \end{array}$$ (6)

*X* represents the number of cycles used to generate the transmission, which involves detecting the speed and range of the victim's strike. When the mantissa reaches the number 0, the mantissa realizes that it is not the right time to attack its prey (65) in Equation 7.


$$\begin{array}{l} p_{h,g}^{s+1}=\frac{p_{h,g}^{s}+p_{g}^{*}}{2}+V_{t}*c_{th,g}^{s} \end{array}$$ (7)

It indicates the location of the prey and is used to reduce the distance between them and speed up the attack process. A mantissa changes its orientation in response to the exchange of a pair of mantissas randomly selected from the entire population (66) in Equation 8.


$$\begin{array}{l} p_{h,g}^{s+1}=Rv_{12}*\left(p_{m,g}^{s}-p_{n,g}^{s}\right)+p_{h,g}^{s} \end{array}$$ (8)

When the function s evaluates to +1, this term $p_{h,g}^{s+1}$ describes the latest position of the *g*th dimension of the mantissa of *h*, $p_{m}^{s}$ and the two random mantissas, are selected from the current population (67) (mentioned in Equation 9).


$$\begin{array}{l} \begin{array}{l} p_{h,g}^{s+1}=p_{h,g}^{s}+E^{2L}\cdot \cos\left(2L\pi \right)\cdot |p_{h,g}^{s}-{\vec{p}}_{mr,g}^{L}|\\ +\left(2Rv_{13}-1\right)\cdot \left(p_{g}^{U}-p_{g}^{L}\right) \end{array} \end{array}$$ (9)

A lower value of variable (*m*) results in higher exploitation and lower search cost in Equation 10.


$$\begin{array}{l} {\vec{p}}_{h}^{s+1}={\vec{p}}_{h}^{s+1}+{\vec{Rv}}_{16}*\left({\vec{p}}_{h}^{s}-{\vec{p}}_{m}^{s}\right) \end{array}$$ (10)

where ${\vec{p}}_{h}^{s}$ represents the female praying mantissa, ${\vec{p}}_{m}^{s}$ represents the fruit chosen randomly from the population that represents the male used by the female for reproduction and consumption. The male-female partners (mentioned in Equation 11) create a new child using the standard intersection operator of genetic operators (68):


$$\begin{array}{l} {\vec{p}}_{h}^{s+1}=\vec{u}*{\vec{p}}_{h}^{s}+\left(p_{11}^{s}+\vec{Rv_{18}}*\times \left(\vec{p_{h}^{s}}-p_{11}^{s}\right)\right)*\left(1-\vec{u}\right) \end{array}$$ (11)

where ${\vec{p}}_{h}^{s}$ denotes the male, μ is the fraction of the male consumed, and the fraction [cos(2πl)] denotes the freedom of the female to replace the male in the objective process. The employed method of feature reduction by MMS is described in Algorithm 1. Using the MMS algorithm for feature reduction enhances the efficiency and accuracy of downstream classification by refining the feature space, improving training convergence, and enabling lightweight models such as LCNN, to make more accurate diagnostic predictions. Not only does this enhance model performance by removing noisy or redundant features, but it also accelerates training and reduces computational expenses, enabling the classifier to function efficiently without sacrificing accuracy.

Algorithm 1 Feature reduction using MMS. 
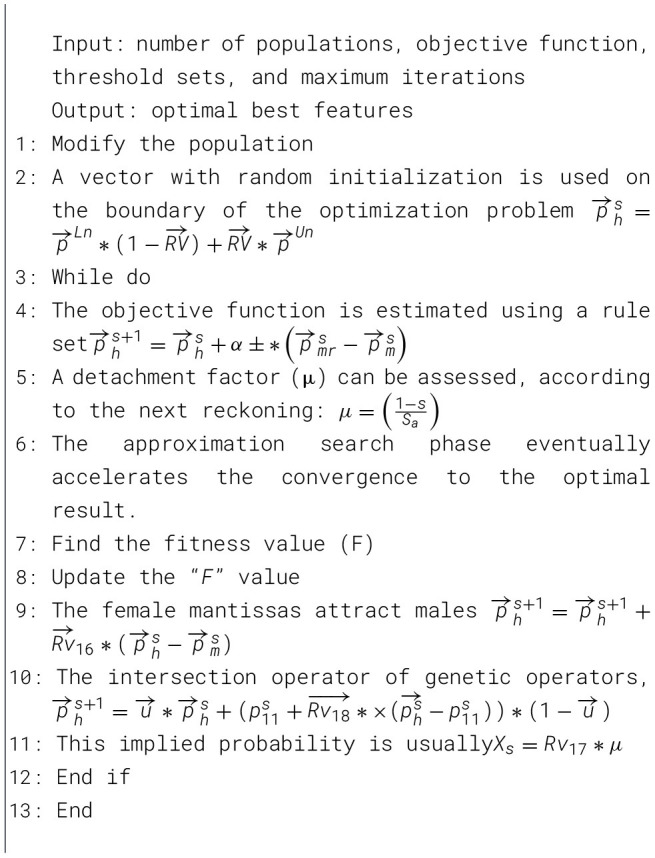

### 3.5 Feature fusion

Multimodal medical image analysis using feature fusion that seeks to combine supplementary data of various types of data (histology and mammography images) to produce a more informative feature representation. By combining the advantages of each modality, feature fusion enhances diagnostic power and robustness in the most daunting tasks, such as breast cancer classification. Nonetheless, one of the issues that most multimodal fusion systems face is the missing modality problem, where a single modality of data could be missing or be incomplete. To address this, the American zebra optimization (AZO) algorithm is adopted as a successful feature fusion strategy. AZO is a metaheuristic algorithm based on nature that replicates the migration and defense behavior of American zebras when environmental changes occur (69). Here, once individual features are identified in both the histology and mammography images and reduced with the MMS algorithm, there is a search for an optimal fusion strategy with the AZO algorithm. This entails selecting the most suitable subset of features for each modality and intelligently combining them to create a strong fused feature vector. It optimizes the various combinations of fusions based on a fitness function, usually classification, to determine which combination of multimodal features is most synergistic (70–72). They have a life cycle that has separate phases, such as group formation, mate separation, migration, dominance establishment, and resource allocation. American Zebra Optimization Algorithm (AZOA) models such behaviors to solve global optimization problems that mimic the coordination, assignment of roles, and migration among the Meta resources of the zebras (e.g., water bodies) in the same way that feature fusion searches for the best solution. Dominant zebras help the other people to orient toward superior solutions, maintain diversity and exploration, and social boundaries discourage inbreeding and improve convergence. Youthful zebras struggle to get fresh grass and green leaves, and therefore, they rely on the pinnacle of the family. We are told about the feeding of American zebras as follows mentioned in Equations 12 and 13 (73).


$$\begin{array}{l} {\bar{W}}_{h}^{g}=\\ \{\begin{array}{l} 2r_{1}\sin\left(2\pi r_{2}\right)\times \left(W_{T}^{g}-W_{h}^{g}\right)+W_{T}^{g},ifr_{3}<0.5;\\ 2r_{1}\cos\left(2\pi r_{2}\right)\times \left(W_{T}^{g}-W_{h}^{g}\right)+\;otherwise, \end{array}\forall h\in B_{g} \end{array}$$ (12)

$$\begin{array}{l} W_{h}^{g}=\{\begin{array}{l} {\bar{W}}_{h}^{g},\;{\bar{f}}_{h}^{g}<f_{h}^{g}\\ W_{h}^{g},otherwise, \end{array} \end{array}$$ (13)

In AZOA algorithm, the mare is represented by $W_{T}^{g}$ and symbolizes the *h*th zebra of the *g*th set, separately, *B*_*g*_ symbolizes the participants in the *g*th set, *r*_1_ directs even casual worth among [−2, 2] that brings the nourishing of zebra at several slants of 360 marks about the lead of the set, *r*_2_ indicates the flexible variable that is assessed, *r*_3_ indicates an integer between zero and one, the sin and cos activities aid the endeavor of other *h*th associates from multiple perspectives within the clan's head, ${\bar{f}}_{h}^{g}$ is the health score of the *h*th zebra, and finally, ${\bar{W}}_{h}^{g}$ indicates a fresh clinging location when eating mentioned in Equation 14 (74).


$$\begin{array}{l} r_{2}=1-s\times \left(\frac{1}{S}\right) \end{array}$$ (14)

The extreme repetition and the existing repetition are indicated here by S and s. The reproductive function of zebra fish is given as follows in Equations 15 and 16 (75).


$$\begin{array}{l} W_{g}^{y}=crossover\left(W_{h}^{m},W_{g}^{n}\right)\;ifR<xd,h\neq g \end{array}$$ (15)

$$\begin{array}{r} W_{K}^{y}=crossover\left(W_{h}^{m},W_{K}^{d}\right)\;ifR\geq xd,h\neq K\\ \forall h,g,K\in B \end{array}$$ (16)

where $W_{h}^{m}$ depicts the predicament of the *h*th collection's small zebra, $W_{g}^{n}$ means point of zebra b from *g*th set, $W_{K}^{d}$ embodies the point *h*th objective function, and $W_{K}^{y}$ are the situations of zebra *y* in the *g*th set and *K*th collection, individually. A set of zebras, tougher than the rest, maintains the optimal solution by fixing the thresholds of maximum rate of change in Equations 17 and 18 (76).


$$\begin{array}{l} {\bar{W}}_{T}^{g}=\{\begin{array}{l} 2r_{4}\sin\left(2\pi r_{5}\right)\times \left(Zr-W_{T}^{g}\right)+Zr,\;ifr_{6}<0.5;\\ 2r_{4}\cos\left(2\pi r_{5}\right)\times \left(Zr-W_{T}^{g}\right)+Zr,\;otherwise, \end{array} \end{array}$$ (17)

$$\begin{array}{l} W_{T}^{g}=\{\begin{array}{l} {\bar{W}}_{T}^{g},\;{\bar{f}}_{T}^{g}<f_{T}^{g}\\ W_{T}^{g},\;otherwise, \end{array} \end{array}$$ (18)

where *r*_4_ characterizes random numbers in [−2, 2], *r*_5_ indicates the flexible limitation which is resolute, *r*_6_ symbolize sun varying Radom numbers in [0, 1], Zr designates the aquatic capitals, is the *g*th collection lead horse existing location, $W_{T}^{g}$ is the *g*th set lead mare following location, and ${\bar{f}}_{T}^{g}$ is its capability worth of pony in *g*th set (77) in Equation 19.


$$\begin{array}{l} r_{5}=1-s\times \left(\frac{1}{S}\right) \end{array}$$ (19)

In any case, if the leader in the collection develops weak, it is necessary to vary the lead. The leadership transition phase is used to elect a new leader (78) in Equation 20.


$$\begin{array}{l} W_{T}^{g}=\{W_{h}^{g},iff\left(W_{h}^{g}\right)<\left(W_{T}^{g}\right),\forall h\in B_{g} \end{array}$$ (20)

where $W_{T}^{g}$ characterizes *j*th set leads' horse existing location and is the capability worth of the spearhead stallion. The AZO algorithm simulates zebra behaviors, such as migration toward optimal resources and social structures, to ensure diversity and cooperation (Algorithm 2). In feature fusion, these behaviors help select and combine the best features from histology and mammography images. The living arrangement of the zebra, whereby the strongest ones lead, is similar to the mechanism of determining the synergistic properties so as to achieve better classification. All zebra solutions are attracted to superior feature subsets to enable a comprehensive search over the fusion space. The social boundaries do not allow convergence prematurely; diversity is preserved in the search for features.

Algorithm 2 Feature fusion using AZO. 
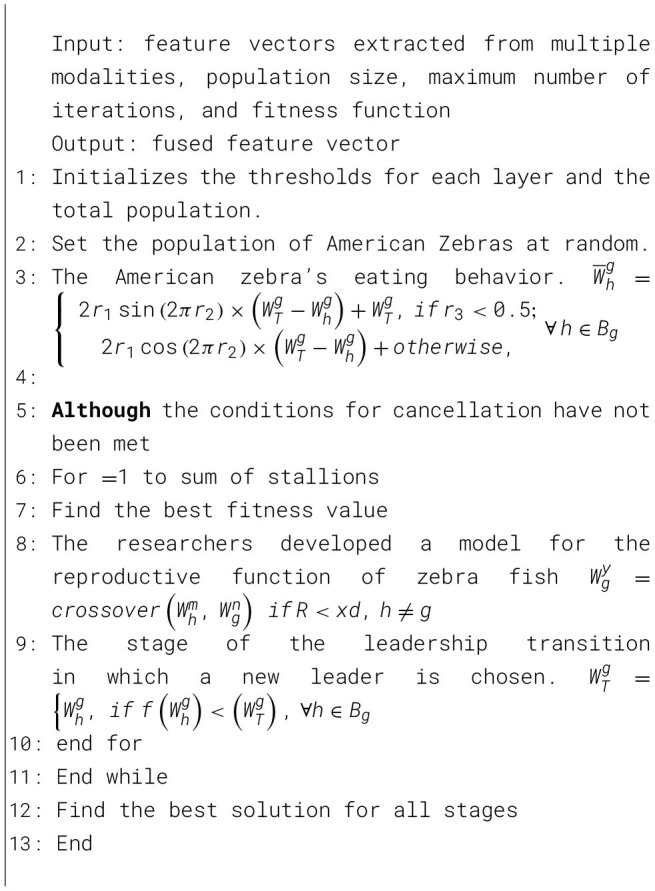

### 3.6 Detection and classification of breast cancer

Detection aims to identify possible tumor areas, whereas classification identifies the type of lesion. It is important to note that this process is essential to make a timely diagnosis that greatly enhances treatment outcomes and survival rates (79). In this regard, a lightweight convolutional neural network (LCNN) is employed because it is efficient and incurs less computational power. LCNN is a smaller implementation of regular CNN models (80), with fewer parameters and layers but still achieving high accuracy. Here, the LCNN accepts the optimized fused features, which were previously obtained in the preprocessing phase, feature extraction phase, feature reduction phase, and fusion, and subjects them to the convolutional layers, which are used to extract spatial hierarchies of patterns. It uses a depth-wise separable convolution layer as the first stage, which takes preprocessed mammography and histology images, followed by convolution layers. The training is made more stable by batch normalization, whereas non-linearity is added by Rectified Linear Unit (ReLU) activation. Maximum pooling reduces spatial dimensionality, and dropout mitigates overfitting (81). The last layers are used to classify them into certain groups that include benign, malignant, and particular types of breast cancer. Once the pictures have been shrunk by the convergence layer, less computational power is utilized in the subsequent steps in Equation 21 (82).


$$\begin{array}{l} V_{d}=P*\omega_{d}=\overset{b}{\underset{K=1}{\sum }}P^{K}*\omega_{d}^{K} \end{array}$$ (21)

The variable b characterizes the number of input matrices, *P* = [*P*^1^, *P*^2^, ...*P*^*b*^], is the *k*th participation matrix, ω_*d*_ is the intricacy kernel of filter d, $\omega_{d}=\left[P_{d}^{1},P_{d}^{2},...P_{d}^{b}\right]$, is the medium of the *k*th sub-convergence seed of the convergence grain, and * designates the convergence procedure mentioned in Equation 22 (83).


$$\begin{array}{l} \begin{array}{l} o_{d}=\frac{V_{d}-e\left[V\right]}{\sqrt{\mathrm{var}\left[V\right]+\varepsilon }}\times \gamma_{d}+\beta_{d}\\ =\frac{\overset{b}{\underset{K=1}{\sum }}\left(P^{K}*\omega_{d}^{K}\right)-\mu_{d}}{\sigma_{d}}\times \gamma_{d}+\beta_{d}\\ =\overset{b}{\underset{K=1}{\sum }}\left(P^{K}*\frac{\gamma_{d}}{\sigma_{d}}\omega_{d}^{K}\right)-\frac{\mu_{d}\gamma_{d}}{\sigma_{d}}+\beta_{d}\\ =P⊗\omega_{d} \end{array} \end{array}$$ (22)

where ⊗ denotes the convolution process, μ_*d*_ = *e*[*V*]D, $\sigma_{d}=\sqrt{\mathrm{var}\left[V\right]+\varepsilon }$, γ, and β are the learned rule constants and bias relations, both of which are trainable limits, and e[V] and Var[V] are the error and adjustment of the essentials in direction v, separately. The mesh has the following activation function (Equation 23) (84).


$$\begin{array}{l} mish\left(p\right)=p\times \tan i\left(\ln\;\left(1+E^{p}\right)\right) \end{array}$$ (23)

Batch Normalization (BN) may fuse homogeneously to a convolution with extra bias because of its homogeneity. It is evident that $-\frac{\mu_{d}\gamma_{d}}{\sigma_{d}}+\beta_{d}$ may be constructed as an extra bias term for $\frac{\gamma_{d}}{\sigma_{d}}\omega_{d}$ for every branch, if a similar productivity is needed for the neural network core ω_*d*_. The convolutional layer is where the two BN branches combine (85). Set ${\bar{\omega }}_{d}$ and ${\hat{\omega }}_{d}$ to be the grains of the conforming riddles in the 3 × 3 intricacy and *K* × *K* complication layers, separately, and to be the deep, large neural network kernel following fusion, to be the gained bias term for each filter *d* in Equations 24, 25 and 26 (86).


$$\begin{array}{l} \omega_{d}=\frac{{\bar{\gamma }}_{d}}{{\bar{\sigma }}_{d}}{\bar{\omega }}_{d}⊕\frac{{\hat{\gamma }}_{d}}{{\hat{\sigma }}_{d}}{\hat{\omega }}_{d} \end{array}$$ (24)

$$\begin{array}{l} n_{d}=\frac{{\bar{\mu }}_{d}{\bar{\gamma }}_{d}}{{\bar{\sigma }}_{d}}-\frac{{\hat{\mu }}_{d}{\hat{\gamma }}_{d}}{{\hat{\sigma }}_{d}}+{\bar{\beta }}_{d}+{\hat{\beta }}_{d} \end{array}$$ (25)

$$\begin{array}{l} {ō}_{d}=\overset{b}{\underset{K=1}{\sum }}\left(P^{K}*\frac{{\bar{\gamma }}_{d}}{{\bar{\sigma }}_{d}}{\bar{\omega }}_{d}^{K}\right)-\frac{{\bar{\mu }}_{d}{\bar{\gamma }}_{d}}{{\bar{\sigma }}_{d}}+{\bar{\beta }}_{d}=P⊗{\bar{\omega }}_{d} \end{array}$$ (26)

where ⊕ is the initial sum of kernel limits in the same conditions, that is, the smaller kernel merges with the larger kernel. ${\bar{o}}_{d}$ and ô_*d*_ are the outputs of the branches of the 3 × 3 complication and the creative *K* × *K* complication, separately. The *c*th element in the output of the compression function z is defined as follows in Equation 27 (87).


$$\begin{array}{l} w_{d}=f_{ty}\left(V_{d}\right)=\frac{1}{I\times Z}\overset{i}{\underset{h=1}{\sum }}\overset{Z}{\underset{g=1}{\sum }}V_{d}\left(h,g\right) \end{array}$$ (27)

where $v_{d}\in r^{I\times Z}$, *V* = [*V*_1_, *V*_2_, ...*V*_*d*_]. The output of transform *V* can be understood as a set of local descriptors whose statistical properties can be generalized to cover the entire image. The final output is obtained by rescaling v with the beginnings in Equation 28 (88).


$$\begin{array}{l} {\tilde{p}}_{d}=f_{Scale}\left(v_{d},t_{d}\right)=t_{d}v_{d} \end{array}$$ (28)

where the channel-wise development among the feature mapping *v*_*d*_ and the scalar *t*_*d*_ is indicated by and *f*_*Scale*_. The network can use global information to intelligently highlight relevant information features while suppressing irrelevance (89). The information gathered from the collection and the initial tags that accompanied it are here, and the *j*th classifying neuron's result indicates that the example belongs to the *g*th classification in Equation 29 (90).


$$\begin{array}{l} X\left(q_{b}=g|p_{b};\theta \right)=\frac{Exp\left(\phi_{\theta^{*}}{\left(p_{b}\right)}^{S}Z_{ta}^{\left(g\right)}+n_{ta}^{\left(g\right)}\right)}{\sum_{K=1}^{K}Exp\left(\phi_{\theta^{*}}{\left(p_{b}\right)}^{S}Z_{ta}^{\left(K\right)}+n_{ta}^{\left(K\right)}\right)} \end{array}$$ (29)

$\phi_{\theta^{*}}\left(\cdot \right)$ is the structure removed from all covers, θ^*^ is the restrictions of all features deposits excluding the classifier, and $\theta =\left\{\theta^{*},\theta_{ta}\right\}$ is all potential network settings that can be trained (91). The calculation method of the trial *p*_*b*_ is to exploit the later possibility, that is mentioned in Equation 30,


$$\begin{array}{l} {\tilde{q}}_{b}=Arg\max X\left(q_{b}=g|p_{b};\theta \right) \end{array}$$ (30)

Throughout the exercise stage, the cross-entropy (CE) loss purpose inside the system, cross-wise the whole of the objective function (Equation 31) (92).


$$\begin{array}{l} G\left(\theta \right)=-\frac{1}{B}\overset{B}{\underset{b=1}{\sum }}\overset{k}{\underset{g=1}{\sum }}H\left\{q_{b}=g\right\}\ln\;X\left(q_{b}=g|p_{b};\theta \right) \end{array}$$ (31)

where *B*; *H*{·} is an indicator function, *H*{true} = 1, and *H*{false} = 0. Algorithm 3 outlines how LCNN works for cancer diagnosis. Algorithm 3 describes the working process of LCNN for cancer detection. The residual connections form a deep architecture of ResNet, which results in a high number of parameters and computational cost, and the dense connections of DenseNet, which results in high resource demands. Compared to LCNN, the fewer number of layers and separable convolutions in LCNN are employed to reduce both the number of parameters and computational complexity, leading to a shorter inference time and lowering memory consumption, thus making LCNN best suited to a real-time application with resource constraints. It has a shorter training period, which is more applicable to mobile or embedded medical devices, and still achieves high-classification accuracy.

Algorithm 3 Cancer detection using LCNN. 
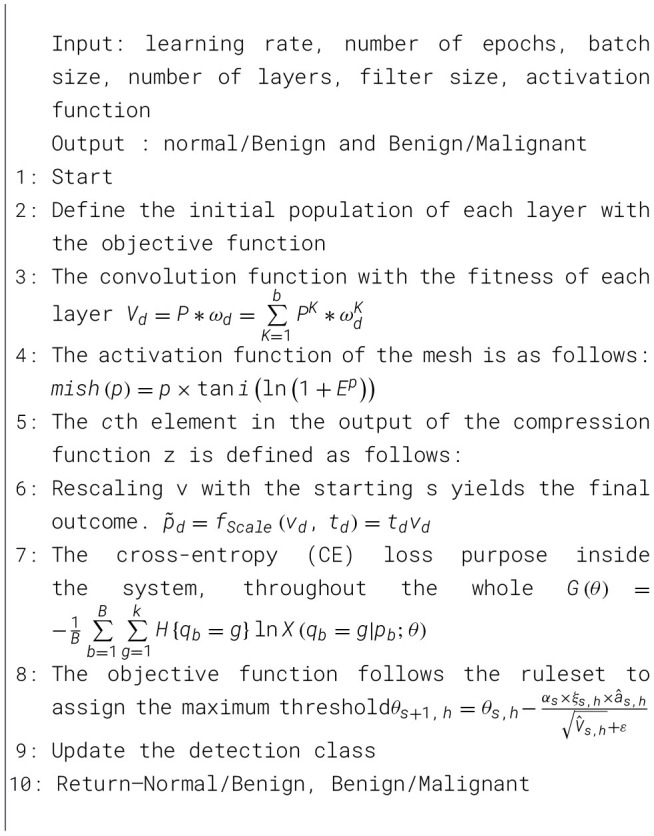

## 4 Results analysis

The results and comparison between the proposed model's effectiveness and the existing models in different simulation scenarios are provided. The benchmark MIAS and BreakHis datasets have been used to validate the effectiveness of the method. The outline and algorithmic processes were implemented in Python using libraries including TensorFlow, Keras, NumPy, and Matplotlib. It was first run on the Google Colaboratory (Google Colab) platform, which was supported by a Google Compute Engine with a backend that is compatible with a graphics card. Such augmentations were random rotations, horizontal and vertical flips, scaling, and brightness. Only the training set could be augmented to avoid data leakage into either validation or testing phases, so that performance measures could indicate the model's actual ability on unseen data.

### 4.1 Results analysis of feature extraction models

**Figure 3:**
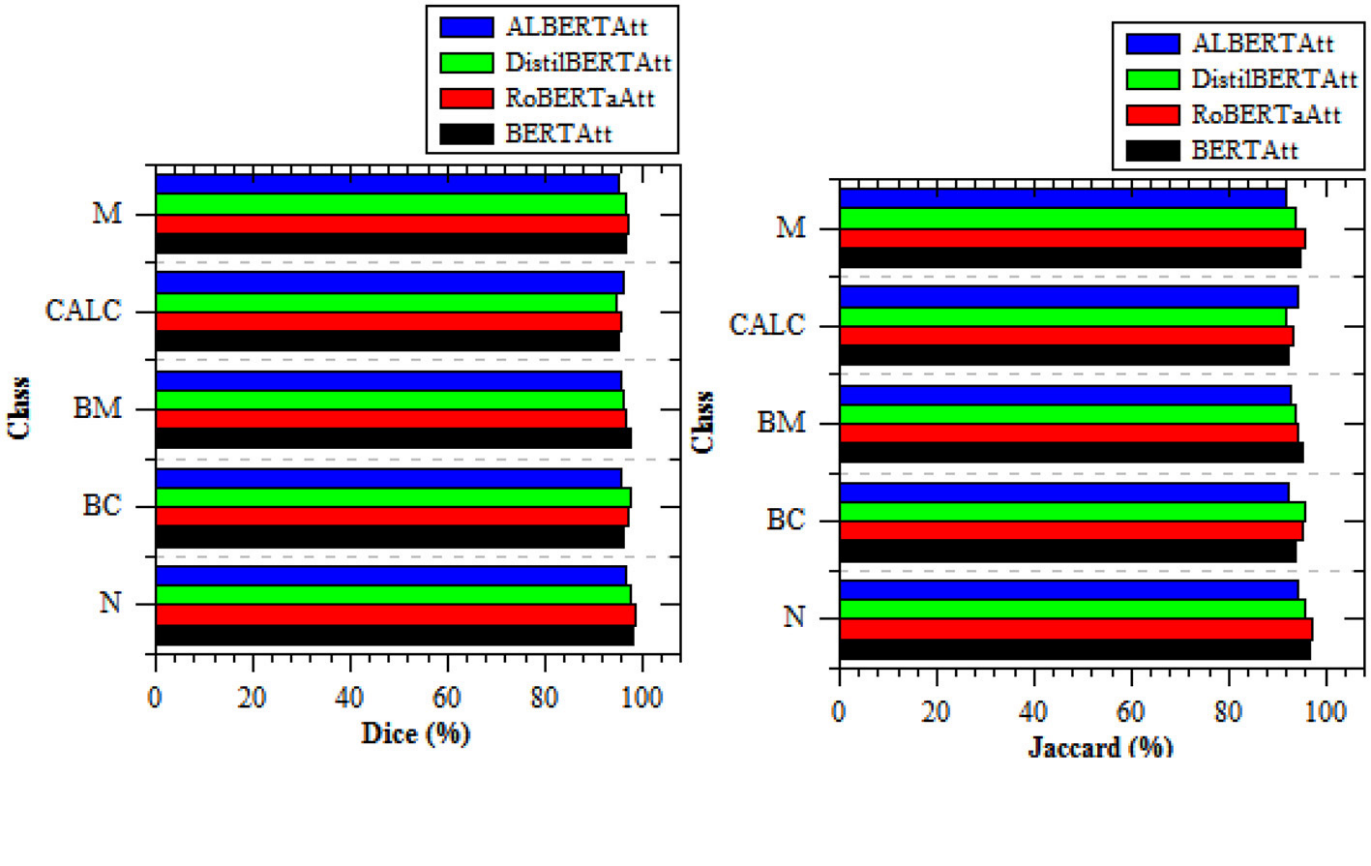
Results comparison of proposed models for feature extraction. **(a)** Dice similarity index; **(b)** Jaccard index on MIAS dataset.

**Figure 4:**
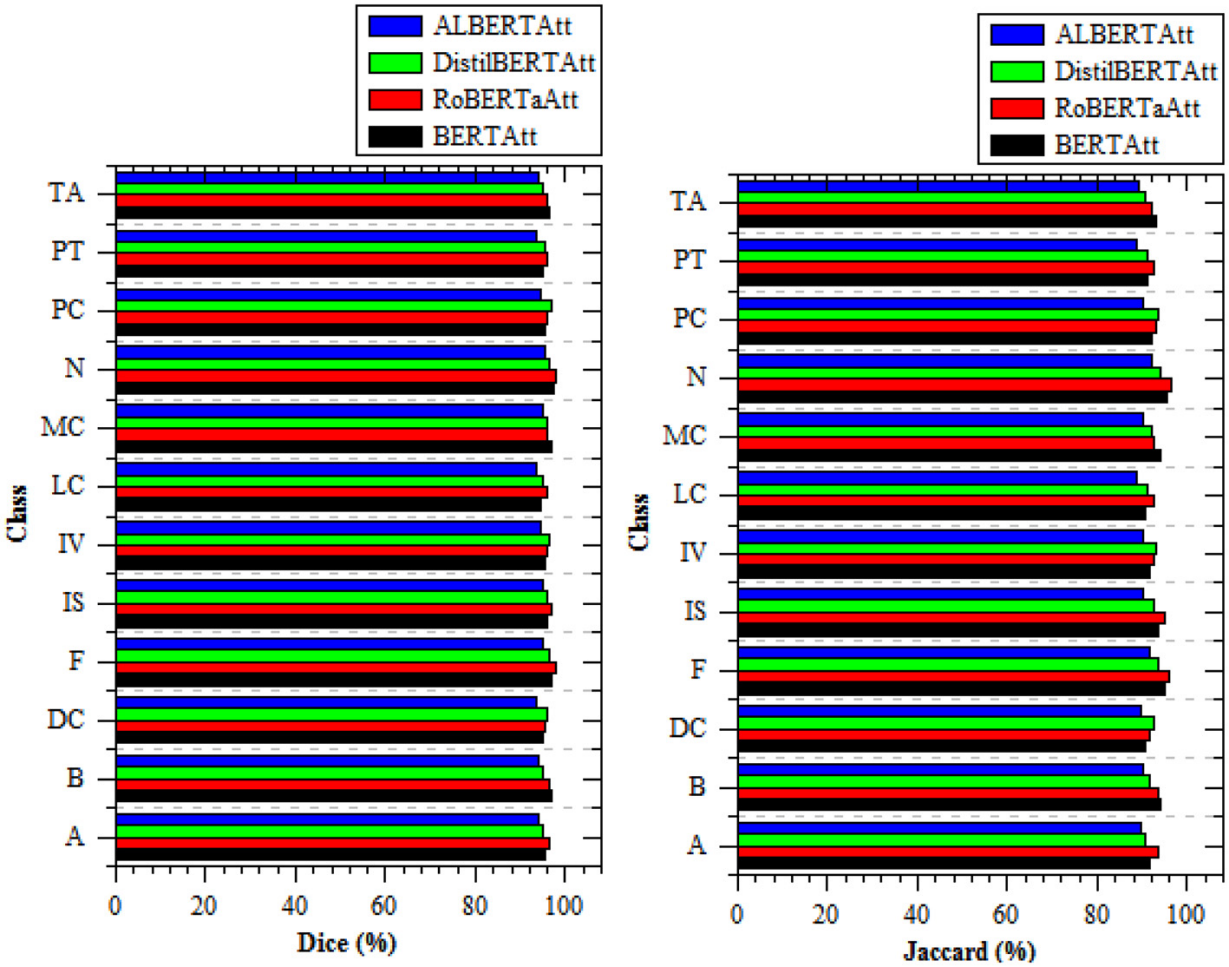
Results comparison of proposed models for feature extraction. **(a)** Dice Similarity Index; **(b)** Jaccard Index on BreakHis dataset.

The feature extraction model results that comprise BERTAtt, RoBERTaAtt, DistilBERTAtt, and ALBERTAtt are examined with regard to *k*-fold cross-validation. The result of the presentation comparison of four transformer-based attention replicates, the MIAS dataset with the Dice Similarity Index, and Jaccard Index, as indicated in Figure 3, demonstrates informative trends. When comparing the BreakHis dataset with 12 histology subtypes using the Dice Similarity Index and the Jaccard Index, it is clear that RoBERTaAtt achieves the highest, or nearly the highest, scores in the majority of the classes. In other classes, ALBERTAtt has shown comparatively lower Dice scores, losing to RoBERTaAtt by 1.232%, BERTAtt by 0.502%, and ALBERTAtt by 2.15%, indicating that it is considerably less capable of localizing features at the pixel level in histology images. In the majority of the categories, DistilBERTAtt has shown a superiority of 0.502% over BERTAtt, which is 0.502% less than RoBERTaAtt; this observation also applies to the other classes. The normal class had an increase of 0.927% over BERTAtt and an even higher increase of 2.101% over DistilBERTAtt. DistilBERTAtt achieved the largest Jaccard score of 93.765% in the PC class, an increase of 0.782% over RoBERTaAtt and 1.616% BERTAtt. ALBERTAtt had always had lower Jaccard values, lagging behind RoBERTaAtt by 2.5–3.2% in a few classes, including LC, PT, and TA, once again confirms its lower segmentation overlap precision (Figure 4). RoBERTaAtt had the best and most balanced results in the BreakHis data, both in Dice and Jaccard indices.

### 4.2 Impact of feature reduction

Table 3 shows the effect of the proposed model on feature reduction across three large datasets, namely, MIAS, BreakHis, and a custom multimodal dataset. The findings reveal that incorporation of the MMS based feature reduction is a major contributor to the discriminative ability of the transformer-based models like BERTAtt, RoBERTaAtt, DistilBERTAtt, and ALBERTAtt. In the MIAS dataset, the classification accuracy had significant improvement in all classes, with the majority of the models achieving improvement of up to 3%. As an example, the precision of the malignant category with RoBERTaAtt increased from 94.827 to 97.872%, indicating the well-generated feature space achieved with the help of MMS. The same level of performance increase can be observed in the BreakHis dataset, especially in the hard classes such as MC and TA, where the MMS-enhanced models achieved by up to 3.5% higher accuracy.

**Table 3:** Results of with and without feature reduction on MIAS, BreakHis, and multimodality datasets.

Class	Without MMS algorithm				With MMS algorithm			
	BERTAtt	RoBERTaAtt	DistilBERTAtt	ALBERTAtt	BERTAtt	RoBERTaAtt	DistilBERTAtt	ALBERTAtt
MIAS dataset								
N	94.312	94.918	94.021	94.087	97.218	97.945	97.004	96.781
BC	94.415	94.702	94.335	94.103	97.304	97.836	97.123	96.911
BM	94.689	94.991	94.578	94.266	97.438	97.984	97.305	97.029
CALC	94.081	94.383	94.012	94.096	97.104	97.726	96.963	96.738
M	94.598	94.827	94.452	94.209	97.351	97.872	97.143	96.981
BreakHis dataset								
A	94.219	94.618	94.088	94.022	97.178	97.744	96.849	96.512
B	94.302	94.727	94.163	94.054	97.309	97.803	97.098	96.784
DC	94.445	94.916	94.307	94.101	97.426	97.926	97.241	96.882
F	94.618	94.995	94.382	94.278	97.504	98.061	97.374	97.142
IS	94.036	94.504	94.001	94.025	97.016	97.583	96.724	96.391
IV	94.285	94.762	94.212	94.038	97.278	97.819	97.083	96.789
LC	94.489	94.847	94.308	94.149	97.401	97.921	97.215	96.976
MC	94.663	94.991	94.487	94.333	97.523	98.044	97.397	97.163
N	94.712	95.003	94.532	94.397	97.598	98.072	97.502	97.194
PC	94.386	94.742	94.145	94.032	97.328	97.896	97.059	96.701
PT	94.113	94.589	94.008	94.061	97.105	97.643	96.771	96.428
TA	94.652	94.993	94.414	94.255	97.509	98.067	97.358	97.126
Multimodal dataset								
Normal	95.029	95.412	94.814	94.391	98.006	98.633	97.835	97.481
Benign	94.917	95.238	94.705	94.221	97.902	98.514	97.702	97.358
Malignant	94.778	95.073	94.592	94.063	97.781	98.382	97.604	97.122

### 4.3 Results of feature fusion models

Table 4 presents a detailed analysis of the various approaches to feature fusion used on the multimodal breast cancer dataset with transformer-based models and lightweight CNNs. The findings clearly indicate that there is a significant benefit in applying feature fusion techniques such as MMS and AZO to the classification system. The models performed at baseline with no feature fusion had the highest accuracy of RoBERTaAtt + LCNN 94.85%. When using MMS, all models became more accurate. The DistilBERTAtt and ALBERTAtt versions also enjoyed the same advantages with the combined MMS + AZO fusion, with improvements of 5.1 and 5.1% in accuracy, respectively.

**Table 4:** Results comparison of feature fusion models on the multimodality dataset.

Feature fusion	Model	Values in %			
		Accuracy	Precision	Recall	F-measure
Without feature fusion	BERTAtt + LCNN	94.210	93.800	94.500	94.150
	RoBERTaAtt + LCNN	94.850	94.200	94.900	94.550
	DistilBERTAtt + LCNN	93.600	93.000	93.800	93.400
	ALBERTAtt + LCNN	93.900	93.400	94.100	93.750
	BERTAtt + MMS + LCNN	95.800	95.300	96.000	95.650
	RoBERTaAtt + MMS + LCNN	96.300	95.700	96.400	96.050
	DistilBERTAtt + MMS + LCNN	95.100	94.600	95.400	95.000
	ALBERTAtt + MMS + LCNN	95.400	94.800	95.600	95.200
With feature fusion	BERTAtt + AZO + LCNN	97.500	97.100	97.600	97.350
	RoBERTaAtt + AZO + LCNN	98.100	97.700	98.200	97.950
	DistilBERTAtt + AZO + LCNN	97.000	96.600	97.200	96.900
	ALBERTAtt + AZO + LCNN	97.300	96.900	97.400	97.150
	BERTAtt + MMS + AZO + LCNN	99.100	98.800	99.200	99.000
	RoBERTaAtt + MMS + AZO + LCNN	99.860	99.700	99.900	99.800
	DistilBERTAtt + MMS + AZO + LCNN	98.700	98.300	98.900	98.600
	ALBERTAtt + MMS + AZO + LCNN	99.000	98.600	99.100	98.850

### 4.4 Performance of detection and classification models

**Figure 5:**
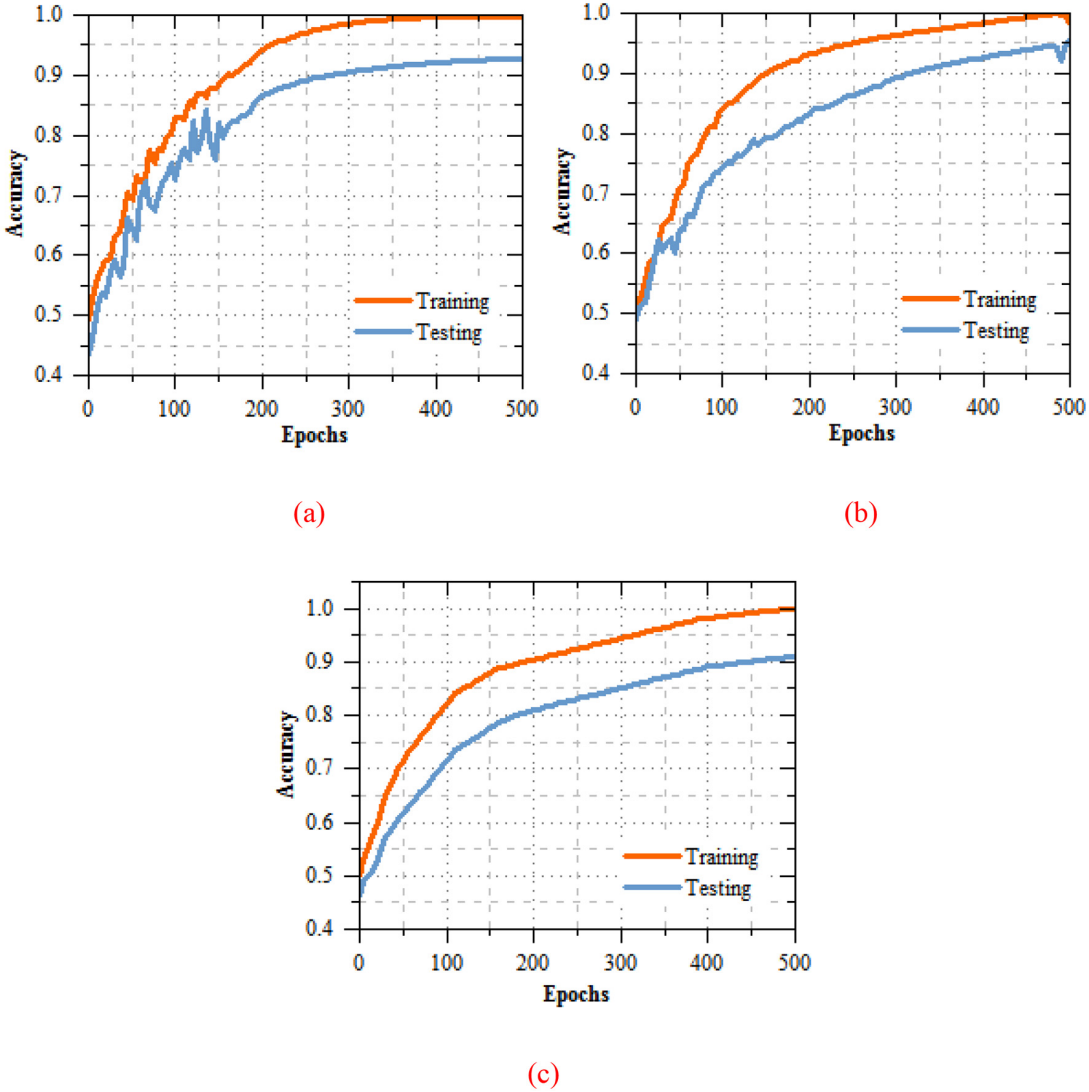
Training and testing accuracy of LCNN using **(a)** MIAS, **(b)** BreakHis, and **(c)** multimodal datasets.

**Figure 6:**
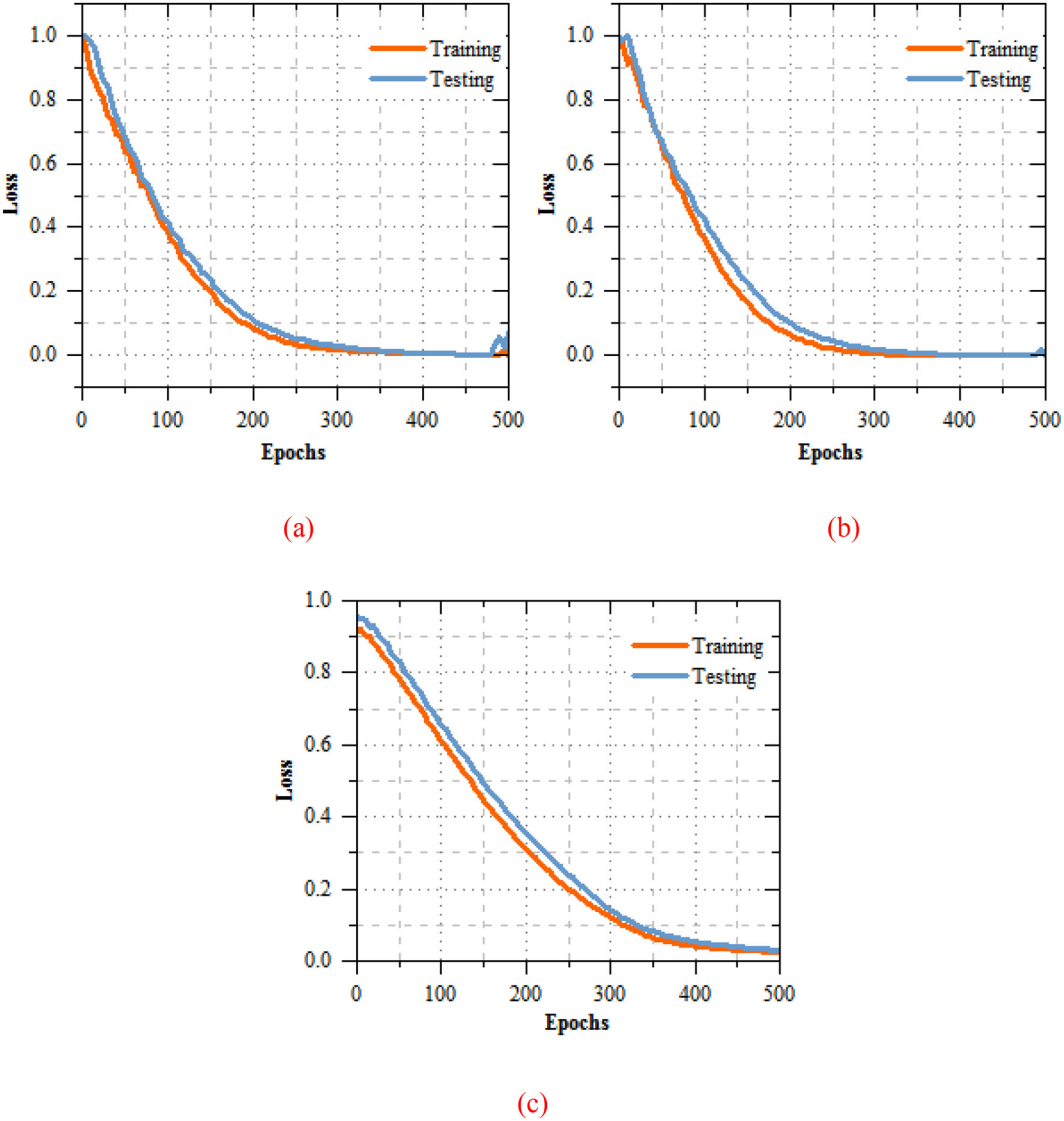
Loss performance of the LCNN model on breast cancer detection using **(a)** MIAS, **(b)** BreakHis, and **(c)** multimodal datasets.

The changes in the trend of increasing accuracy across the MIAS, BreakHis, and multimodal datasets, which are presented in Figures 5, 6 clearly show that the trend of accurate change of the model above 500 epochs is significant and uniform, and, therefore, the model has a strong power of learning breast cancer. The training accuracy on the MIAS-dataset has improved significantly at the 500th epoch, reaching 99.85%, a very impressive 12.43% increase. The BreakHis dataset experienced an improvement of 94.45%, as suggested by an improvement of 98.25%. The multimodal data that integrates the data of more than one source showed an even better performance as the training accuracy increased to 99.75% against 50%, and the improvement was 99.5%. For the MIAS dataset, the testing accuracy is 92.81%, compared to 43.69%, a significant gain of 12.35%. The BreakHis dataset was close behind it with a testing accuracy of 95.42%. The multimodal data again proved to be the best at the start at 46 and 91.1%, and the actual increase in data was 98.04. The trends in training and testing loss of the LCNN model with breast cancer diagnosis on the MIAS, BreakHis, and multimodal data (Figure 6) are a good indicator of the efficiency of the model in terms of learning and throughout the 500 training epochs. The training loss on the MIAS dataset started at 0.991 and gradually dropped to 0 during the 500th epoch, showing a 10% drop in loss.

**Figure 7:**
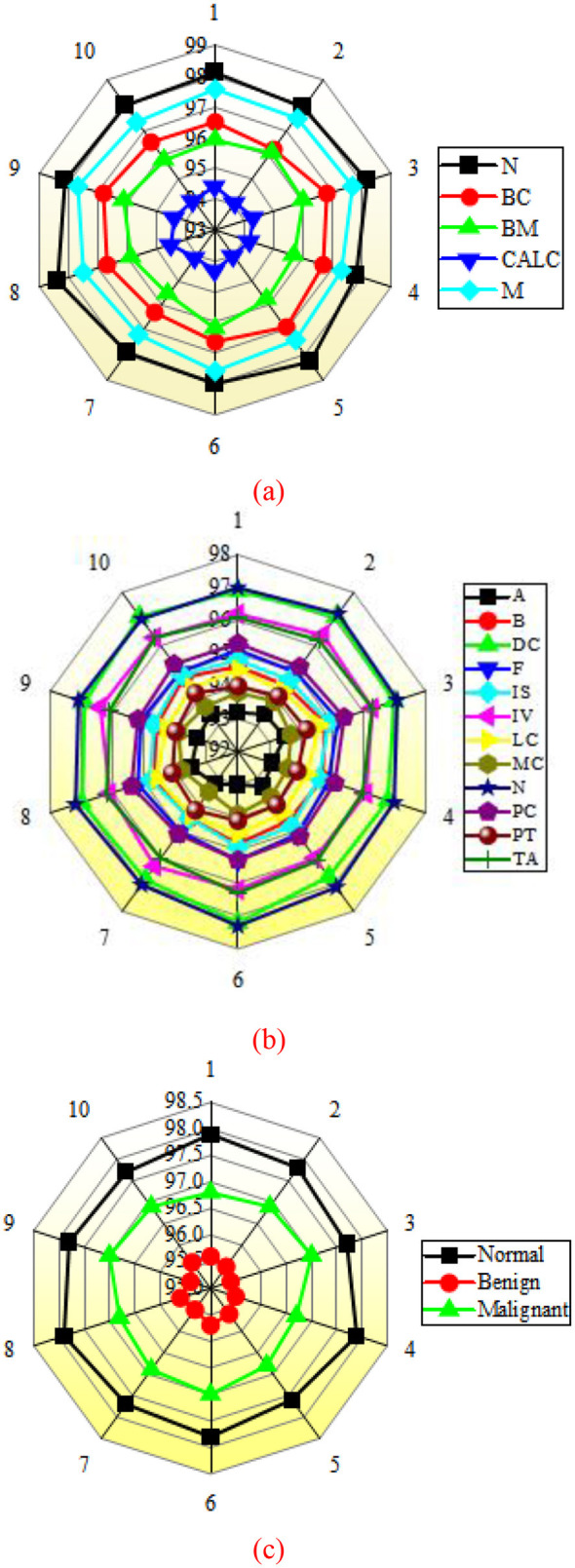
Class-wise accuracy results of LCNN model for breast cancer diagnosis on **(a)** MIAS, **(b)** BreakHis, and **(c)** multimodal datasets over 10-fold cross-validation.

**Figure 8:**
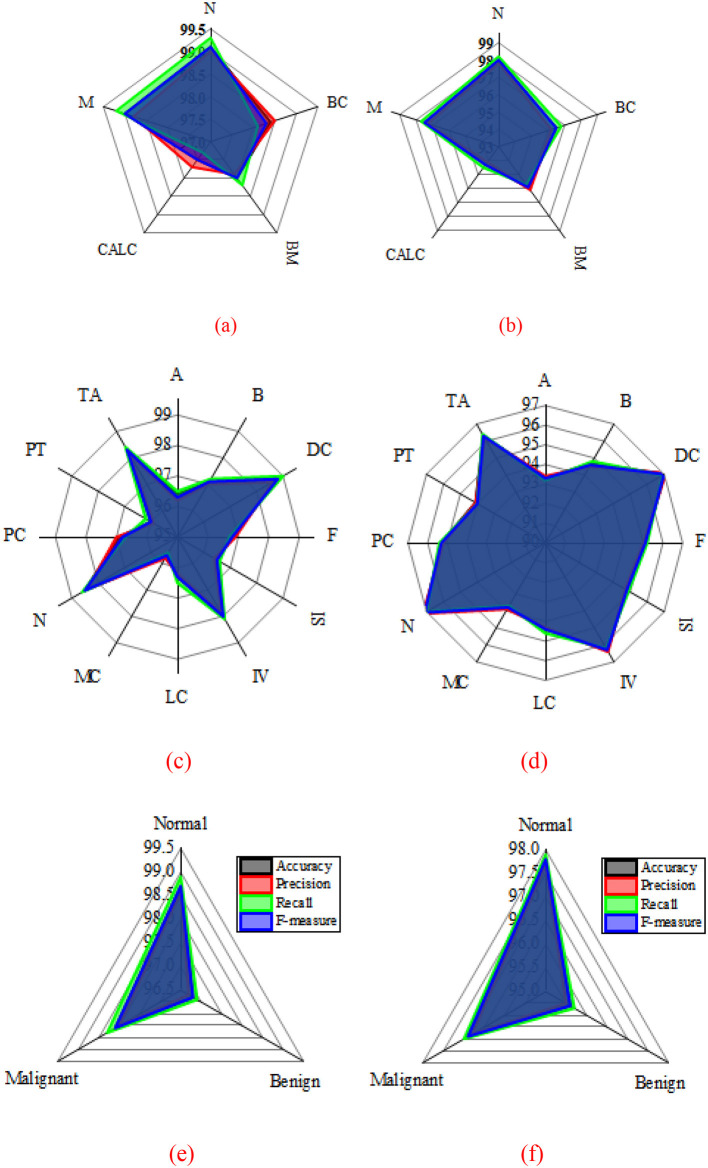
Results of proposed LCNN model for breast cancer diagnosis on **(a, b)** MIAS dataset (testing and testing set); **(c, d)** BreakHis (testing and testing set); **(e, f)** multimodal (testing and testing set) datasets.

**Figure 9:**
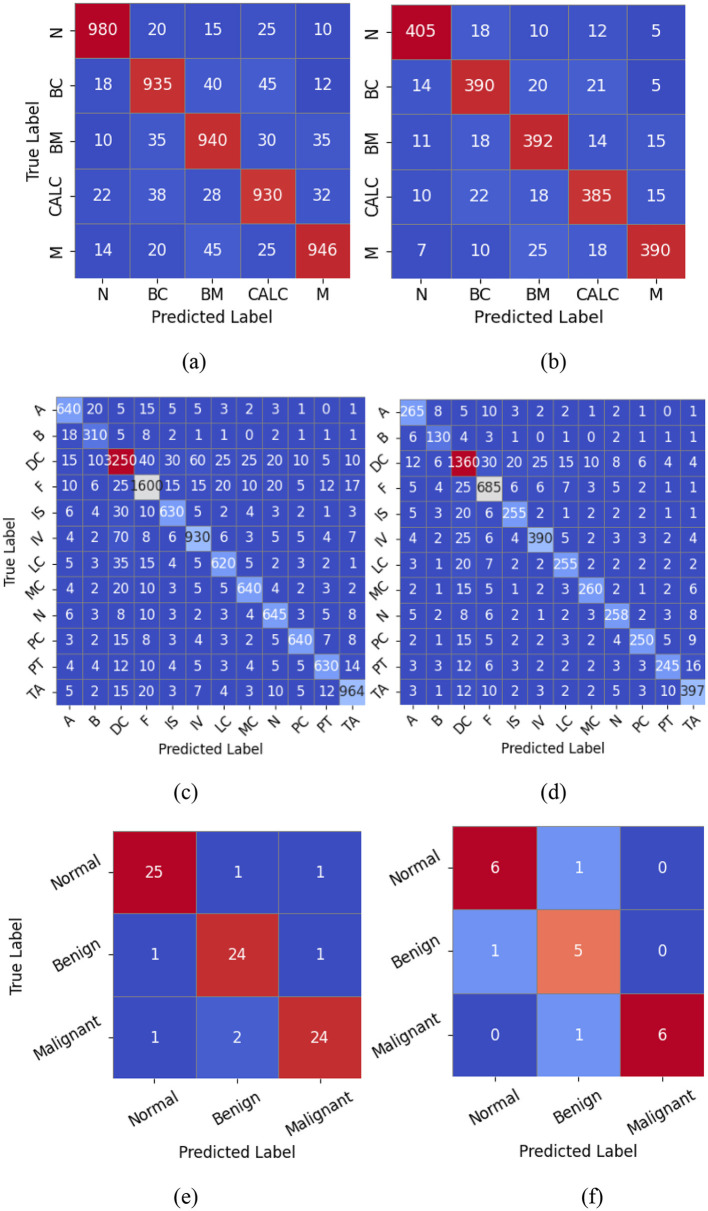
Confusion matrix of proposed LCNN model **(a, b)** MIAS dataset (testing and testing set); **(c, d)** BreakHis (testing and testing set); **(e, f)** multimodal (testing and testing set) datasets.

As stated in Figure 7, the accuracy results of the LCNN model on a class-wise basis from 10-fold cross-validation on the MIAS, BreakHis, and multimodal datasets show the reliable performance of the breast cancer diagnosis model. As shown in Figure 8, the MIAS, BreakHis, and multimodal datasets were used to evaluate the performance of the proposed LCNN model in breast cancer detection (Figure 9). As can be seen in Table 5, the proposed LCNN model is better than both the ResNet and the TwinCNN baseline models in terms of training time and inference time on all modalities. The comparison in Table 6 highlights the suitability of the suggested multimodal model, especially the ALBERTAtt + MMS + AZO + LCNN, which is more effective in a variety of data/sets than the previous approaches. Although the previous literature like Joo et al. (93), Jiang et al. (94), and Misra et al. (95) conducted experiments and demonstrated a maximum accuracy of up to 94.76% with singular modalities such as MRI, ultrasound, and so on, the model has reached a maximum accuracy of 12% and significant performance with a *p*-value of 0.021, which indicate the considerable strength of multimodality over single-modality approaches in the process of early detection of breast cancer (Table 7).

**Table 5:** Performance comparison between the proposed LCNN and baseline models for various modalities.

Modality	Model	Training time (h)	Inference time (s)	Model size (MB)	Memory usage (MB)
Histology	Vision transformer	15.26	3.265	250	450
	EfficientNet	17.859	5.632	312	700
	Multimodal transformer	12.965	2.365	157	398
	LCNN	2.556	0.085	25	200
	ResNet	12.525	0.254	230	800
	TwinCNN	7.876	0.185	150	500
Mammography	Vision transformer	15.478	0.858	300	900
	EfficientNet	21.385	0.745	350	800
	Multimodal transformer	12.78	0.328	250	500
	LCNN	2.345	0.078	25	200
	ResNet	13.256	0.345	230	800
	TwinCNN	8.423	0.247	150	500
Multimodality	Vision transformer	18.347	0.964	350	900
	EfficientNet	13.525	0.754	258	950
	Multimodal transformer	10.636	0.569	190	750
	LCNN	3.152	0.125	30	250
	ResNet	15.068	0.357	230	800
	TwinCNN	9.559	0.229	160	600

**Table 6:** Comparison of SOTA multimodal breast cancer diagnosis studies, highlighting the model architecture, imaging or data modalities used, and reported performance metrics.

Reference	Model	Modalities	Accuracy (mean ±SD)	95% confidence interval
Joo et al. (93)	Two 3D ResNet-50 networks	MRI + clinical	AUC 82.7%	NA
Jiang et al. (94)	Residual + inception CNN	B-mode, elastic ultrasound	94.76%	NA
Misra et al. (95)	Single CNN	B-mode + SE ultrasound	Specificity 94.28%	NA
Kayikci and Khoshgofaar (96)	Sigmoid-gated attention + dense	Text + gene expression + CNA	Accuracy 86.35%	NA
Muduli et al. (97)	Single CNN	Gray-scale (MIAS, DDSM, INbreast)	96.55%, 90.68%, 91.28%	NA
Oyelade et al. (41)	TwinCNN + binary opt	Histology + grayscale	97.7%, 91.3%, 68.4%	NA
This study	BERTAtt + MMS + AZO + LCNN	Histology	98.50 ± 0.17%	[98.36%, 98.64%]
		Mammography	96.67 ± 0.18%	[96.51%, 96.83%]
		Multimodal	99.12 ± 0.13%	[99.00%, 99.24%]
This study	RoBERTaAtt + MMS + AZO + LCNN	Histology	98.65 ± 0.17%	[98.51%, 98.79%]
		Mammography	96.85 ± 0.19%	[96.68%, 97.02%]
		Multimodal	99.25 ± 0.13%	[99.13%, 99.37%]
This study	DistilBERTAtt + MMS + AZO + LCNN	Histology	98.74 ± 0.14%	[98.63%, 98.85%]
		Mammography	97.12 ± 0.16%	[96.98%, 97.26%]
		Multimodal	99.32 ± 0.10%	[99.23%, 99.41%]
This study	ALBERTAtt + MMS + AZO + LCNN	Histology	98.96 ± 0.13%	[98.85%, 99.07%]
		Mammography	97.37 ± 0.16%	[97.23%, 97.51%]
		Multimodal	99.44 ± 0.09%	[99.35%, 99.53%]

**Table 7:** Statistical analysis of different feature extraction models with MMS + AZO + LCNN for breast cancer prediction.

Model 1	Model 2	Modality	Accuracy (%)					
			Model 1	Model 2	*t*-statistic	*p*-value	Effect size (Cohen's *d*)	Wilcoxon *p*-value
BERTAtt	RoBERTaAtt	Histology	98.500	98.650	−0.640	0.534	0.120	0.672
BERTAtt	DistilBERTAtt	Histology	98.500	98.740	−1.210	0.276	0.170	0.594
BERTAtt	ALBERTAtt	Histology	98.500	98.958	−2.730	0.029	0.450	0.028
RoBERTaAtt	DistilBERTAtt	Histology	98.650	98.740	−0.670	0.513	0.090	0.647
RoBERTaAtt	ALBERTAtt	Histology	98.650	98.958	−2.410	0.039	0.440	0.043
DistilBERTAtt	ALBERTAtt	Histology	98.740	98.958	−2.110	0.048	0.340	0.050
BERTAtt	RoBERTaAtt	Mammography	96.670	96.850	−1.430	0.181	0.150	0.380
BERTAtt	DistilBERTAtt	Mammography	96.670	97.120	−2.090	0.045	0.230	0.039
BERTAtt	ALBERTAtt	Mammography	96.670	97.370	–−2.530	0.024	0.300	0.023
RoBERTaAtt	DistilBERTAtt	Mammography	96.850	97.120	−1.150	0.258	0.140	0.438
RoBERTaAtt	ALBERTAtt	Mammography	96.850	97.370	−1.770	0.091	0.220	0.180
DistilBERTAtt	ALBERTAtt	Mammography	97.120	97.370	−1.460	0.181	0.180	0.352
BERTAtt	RoBERTaAtt	Multimodality	99.120	99.250	−1.220	0.264	0.150	0.440
BERTAtt	DistilBERTAtt	Multimodality	99.120	99.320	−2.050	0.045	0.230	0.048
BERTAtt	ALBERTAtt	Multimodality	99.120	99.438	−2.860	0.021	0.400	0.015
RoBERTaAtt	DistilBERTAtt	Multimodality	99.250	99.320	−1.120	0.278	0.100	0.564
RoBERTaAtt	ALBERTAtt	Multimodality	99.250	99.438	−1.950	0.056	0.270	0.070
DistilBERTAtt	ALBERTAtt	Multimodality	99.320	99.438	−1.560	0.137	0.230	0.245

A comparison of computational metrics for the proposed lightweight and baseline models, such as MobileNetV3 and ShuffleNet, is shown in Table 8 across the histology, mammography, and multimodal datasets. Its findings indicate that all the suggested models make considerable reductions in the parameter count, FLOPs, memory footprint, and inference speed, which is due to their effectiveness and applicability to resource-limited clinical settings. Table 9 shows an ablation study that measures the effect of various modalities, fusion strategies, feature selection, and transformers to predict breast cancer using the MMS + AZO + LCNN framework. Relative to the traditional techniques of concatenation or weighted fusion in the absence of MMS, it can be seen that the AZO + MMS + LCNN model in question outperforms the baseline by 0.22% in accuracy across the modalities, which is a clear consequence of the synergy between the optimal features selective step, adaptive fusion process, and lightweight transformer-based features extraction.

**Table 8:** Computational metrics of lightweight and baseline models across histology, mammography, and multimodal datasets.

Model	Modalities	Parameter count (M)	FLOPs (B)	Memory footprint (MB)	Inference time (s)
MobileNetV3	Histology	2.912	0.219	16.423	6.213
	Mammography	2.912	0.219	16.423	6.215
	Multimodal	2.912	0.219	16.423	6.218
ShuffleNet	Histology	1.034	0.074	10.237	4.127
	Mammography	1.034	0.074	10.237	4.130
	Multimodal	1.034	0.074	10.237	4.133
BERTAtt + MMS + AZO + LCNN	Histology	0.512	0.027	4.531	1.218
	Mammography	0.512	0.027	4.531	1.220
	Multimodal	0.512	0.027	4.531	1.223
RoBERTaAtt + MMS + AZO + LCNN	Histology	0.518	0.028	4.542	1.226
	Mammography	0.518	0.028	4.542	1.228
	Multimodal	0.518	0.028	4.542	1.231
DistilBERTAtt + MMS + AZO + LCNN	Histology	0.507	0.027	4.526	1.214
	Mammography	0.507	0.027	4.526	1.217
	Multimodal	0.507	0.027	4.526	1.220
ALBERTAtt + MMS + AZO + LCNN	Histology	0.505	0.027	4.519	1.212
	Mammography	0.505	0.027	4.519	1.215
	Multimodal	0.505	0.027	4.519	1.218

**Table 9:** Ablation study of proposed models showing modality, fusion strategy, and transformer impact on breast cancer prediction metrics.

Modality	Feature fusion and selection	Transformer	Accuracy (%)	Precision (%)	Recall (%)	F-measure (%)
Histology	LCNN only	BERTAtt	98.500	98.455	98.500	98.478
Mammography	LCNN only	BERTAtt	96.670	96.705	96.670	96.688
Histology + Mammography	Concatenation + LCNN	BERTAtt	98.900	98.953	98.900	98.927
Histology + Mammography	Weighted + LCNN	BERTAtt	99.000	99.050	99.000	99.025
Histology + Mammography	AZO + LCNN	BERTAtt	99.120	99.153	99.120	99.137
Histology + Mammography	AZO + MMS + LCNN	BERTAtt	99.320	99.353	99.320	99.337
Multimodality	AZO + MMS + LCNN	BERTAtt	99.320	99.333	99.320	99.327
Multimodality	AZO + MMS + LCNN	RoBERTaAtt	99.250	99.273	99.250	99.262
Multimodality	AZO + MMS + LCNN	DistilBERTAtt	99.320	99.333	99.320	99.327
Multimodality	AZO + MMS + LCNN	ALBERTAtt	99.438	99.453	99.438	99.445
Multimodality	AZO + MMS + LCNN (high λ)	BERTAtt	99.353	99.364	99.353	99.358
Multimodality	AZO + MMS + LCNN (low λ)	BERTAtt	99.120	99.153	99.120	99.137
Histology + Mammography	AZO + LCNN (no MMS)	BERTAtt	99.100	99.123	99.100	99.112
Multimodality	Concatenation + MMS + LCNN	BERTAtt	99.200	99.222	99.200	99.211
Multimodality	Weighted + MMS + LCNN	BERTAtt	99.280	99.303	99.280	99.291

### 4.5 Failure case analysis

**Figure 10:**
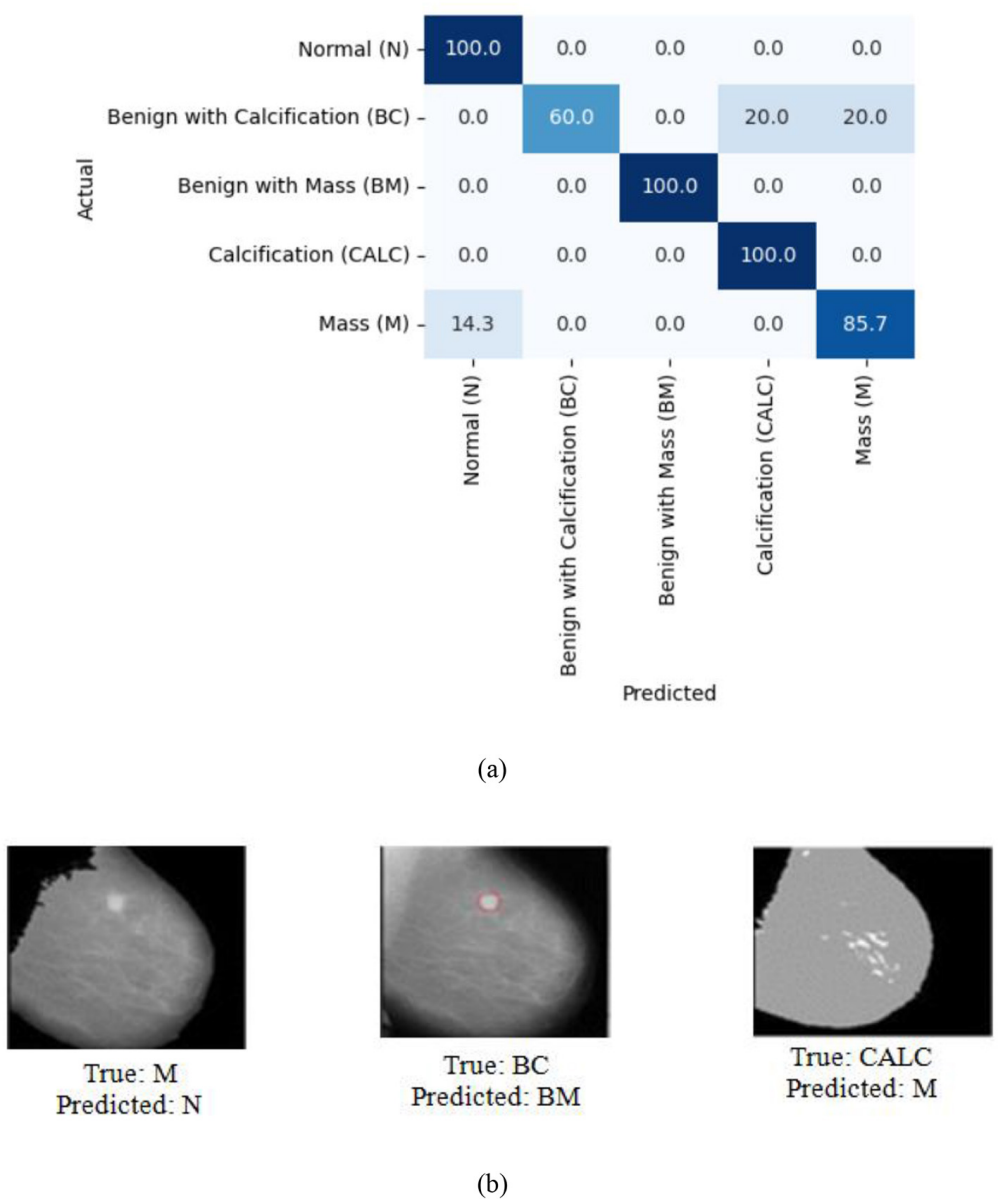
Confusion matrix **(a)** and example misclassified images **(b)** for the MIAS breast cancer dataset. The misclassified images highlight representative cases where the model predicted incorrect classes, illustrating challenging samples and subtle inter-class similarities.

Although the performance of the proposed multimodal framework comprising BERT-based attention, Modified Mantissa Search (MMS), American Zebra Optimization (AZO), and lightweight CNN (LCNN) was strong, several significant failures were observed during the process. These false classifications highlight the existing difficulties in the diagnosis of breast cancer. Low-contrast histology images: there were cases in which poorly differentiated cellular structures or low staining revealed subtle differences between normal and cancerous tissues. The tissue structures around the lesions were also very similar, which increased the difficulty of extracting the features, especially when the lesions were small or embedded. Ambiguity in multimodal fusion: although the AZO-based fusion tended to improve the classification performance, inconsistent results between the histology and mammography tended to generate misleading results for the fusion strategy. Effects of class imbalance: even after extensive rebalancing, the over-augmented training data still had rare malignant subtypes (e.g., mucinous carcinoma, papillary carcinoma in BreakHis) underrepresented. Figure 10 shows the confusion matrix and sample misclassification images of the MIAS data and visualizes these cases of failure.

## 5 Conclusion

The study presents a multimodal breast cancer diagnosis model, featuring attention-based transformers (BERT, RoBERTa, DistilBERT, and ALBERT), which are learned to extract all the features of both histology and mammography images. The model uses the modified mantissa search (MMS) algorithm for dimensionality reduction and the American zebra optimization (AZO) algorithm for robust feature fusion and addressing missing modality data. The lightweight convolutional neural network (LCNN) provides accurate classification, making the model useful in real-world, resource-limited clinical settings. The assessment of the model with the MIAS and BreakHis datasets identifies the model as more efficient than state-of-the-art classifications. The proposed BERTAtt + MMS + AZO + LCNN model (99.12% accuracy) is more accurate than the previous multimodal benchmark models (TwinCNN, 68.4% accuracy) by more than 30 points, and RoBERTaAtt and DistilBERTAtt, as well as ALBERTAtt, are even more successful (99.25, 99.32, and 99.438, respectively).

### 5.1 Limitations and future study

The use of publicly available datasets may not be fully capture the variability and heterogeneity of a real clinical environment. Although the model is effective in managing missing modality data, the performance loss in the presence of several modalities absent or highly corrupted requires further research. The explainability mechanisms are not explicitly included in the current study, which is essential in clinical adoption and trust. To overcome these constraints in future study, there are several pictorial modalities (MRI and ultrasound) to consider; it is better to create explainable parts of AI, and optimize the system to run it on edge and mobile computing. The idea behind such efforts is to bring this research into a practical application as clinical tool that would underpin disease prediction.

## Data Availability

The original contributions presented in the study are included in the article/supplementary material, further inquiries can be directed to the corresponding authors.
